# Inflammation-induced depressed mood and reward responsivity as a function of age in female adults: a randomized controlled trial of endotoxin

**DOI:** 10.1038/s41398-025-03752-2

**Published:** 2025-11-26

**Authors:** Chloe C. Boyle, Joshua H. Cho, Naomi I. Eisenberger, Richard Olmstead, Nina Sadeghi, Daisy Castillo, Michael R. Irwin

**Affiliations:** 1 Norman Cousins Center for Psychoneuroimmunology, UCLA, Los Angeles, US; 2 Jane and Terry Semel Institute for Neuroscience and Human Behavior at UCLA, Department of Psychiatry and Biobehavioral Sciences, David Geffen School of Medicine, Los Angeles, US; 3 Department of Psychology, University of California, Los Angeles, California US

**Keywords:** Human behaviour, Depression

## Abstract

Younger female adults are more vulnerable to depression than older females, potentially due to a greater affective response to inflammation. This randomized, double-blind, placebo-controlled study evaluated depressed mood and reward responsivity in response to an acute inflammatory challenge in younger as compared to older females. Low-dose endotoxin (0.8 ng/kg of body weight) or placebo was administered to younger (n = 40; age 25–44) and older (n = 53; age 60–80) healthy female adults. Participants provided blood samples and self-reported depressed mood pre-infusion and hourly over 9 h. The Effort Expenditure for Rewards Task (EEfRT) and Probabilistic Reward Task (PRT) assessed reward motivation, sensitivity, and learning at baseline and 2.5 h after infusion. Results showed that age moderated the effect of endotoxin on depressed mood (*p* = 0.0005), with endotoxin increasing depressed mood in younger (*p* <0.0001) but not older (*p* = 0.99) females. Age also moderated the effect of endotoxin on EEfRT reward sensitivity (*p* = 0.01), with a decrease in reward sensitivity in younger (*p* = 0.004) but not older (*p* = 0.43) females, with a similar trend observed for EEfRT reward motivation (*p* = 0.09). Age did not moderate the effect of endotoxin on PRT reward learning (*p* = 0.51); endotoxin decreased PRT reward learning in both groups (*p* = 0.04). Results indicate that younger females have heightened sensitivity to the effects of inflammation, resulting in greater increases in depressed mood and larger deficits in reward responsivity compared to older females. Interventions that target inflammation could be relatively more beneficial in the treatment of depression in younger females, as compared to those who are older. Trial Registration: ClinicalTrials.gov: NCT03256760; NCT03848715.

## Introduction

Female sex is associated with an increased risk for depression [1–4], with a markedly higher prevalence in younger as compared to older female adults [5]. The factors underlying this differential risk are poorly understood. Heightened sensitivity to the effects of inflammation may be a key mechanism. Depressed individuals show elevated peripheral inflammation compared to controls according to meta-analyses [6, 7], and inflammation is implicated in depression pathophysiology [6, 8]. For example, immune-based therapies can induce depression [9], and experimental acute inflammatory challenges can induce depressed mood [10]. However, these effects are often variable, suggesting inflammation may be most related to depression responses in a subset of vulnerable individuals [11–14].

Younger females show a robust depressive sensitivity to inflammatory challenge. In experimental models, younger females show greater inflammation-induced depressed mood [13, 15] as compared to age-comparable males. It is possible that sensitivity to inflammation-induced depression may decrease as females age. For example, depression is less prevalent in older than younger females [5], despite aging increasing systemic inflammation (i.e., inflammaging) [16]. Further, one study found that correlations between inflammation and depressive symptoms weaken with age [17]. Despite these findings, no experimental studies have tested the effects of inflammation on depressed mood as a function of age in female adults.

Among the various dimensions of depression, it appears that inflammation is strongly linked to anhedonia (loss of interest or pleasure) [18–21]. Anhedonia reflects deficits in reward processing, including loss of motivation, reduced sensitivity to rewards, and impaired reward learning [22]. Experimental models show that inflammation contributes to reward dysregulation [23–25], inducing anhedonic behavior in preclinical models [26, 27] and reducing neural reactivity to monetary reward in humans [28–30]. Behavioral studies indicate that inflammation alters reward motivation (e.g., willingness to exert effort for reward) [31–33], modulates reward learning (e.g., the ability to learn from and accumulate rewards over time) [30, 33], and inconsistently impacts reward sensitivity (e.g., responsivity to changes in reward magnitude) [23, 31–35]. One study found more robust deficits in inflammation-induced neural response to reward anticipation in younger female as compared to younger male adults [36]. Yet, no studies have evaluated whether inflammation modulates these three reward domains in younger as compared to older females. Given that these reward domains are biologically dissociable and subjectively distinct [23], clarifying whether they are differentially responsive to inflammatory signaling as a function of age has the potential to inform targeted treatment of depression.

To address these questions, this study tests whether experimentally induced inflammation differentially influences the primary outcome, reward processes, and the secondary outcome, depressed mood, in younger as compared to older females. In this double-blind, placebo-controlled inflammatory challenge with endotoxin, younger (25–44 years; n = 40) and older (60–80 years; n = 53) females completed two standard behavioral reward tasks at a baseline visit and at 2.5 h post-infusion, when the inflammatory cytokine response peaks [13], and reported on depressed mood pre-infusion and hourly over 9 h post-infusion. For our primary outcome, we hypothesized that younger females would exhibit greater endotoxin-induced reductions in reward motivation, reward sensitivity, and reward-related learning, as compared to those who were older. Further, consistent with depressed mood responses previously observed [13, 15], we secondarily hypothesized that younger females would exhibit greater increases in endotoxin-induced depressed mood scores in comparison to older females.

## Materials and methods

### Trial design and oversight

This randomized controlled, double blinded, parallel design, single site trial adhered to Consolidated Standards of Reporting Trials (CONSORT). The study was approved by the University of California, Los Angeles (UCLA) Institutional Review Board (IRB approval number: 16-000583) and conducted in accordance with the Declaration of Helsinki. Participants were enrolled in the protocol between August 22, 2017 and November 20, 2022 and provided written informed consent before baseline assessment. Prospective registration of this study at ClinicalTrials.gov involved two registration documents (NCT03256760; NCT03848715). One (NCT03256760) was limited to adults older than 60 years, and the other (NCT03848715) was limited to female adults. The experimental protocol, as previously described [37], was identical for the two clinical trial registration documents, which was approved under a single IRB (16-000583) and funded by the same R01 (AG051944-03; MRI, Principal Investigator), with a related administrative supplement for the study of younger females as compared to older females (AG051944 04S1; MRI, Principal Investigator). An independent Data and Safety Monitoring Board provided semi-annual oversight of the study.

### Participants

The sample (*n* = 93) was comprised of two groups: younger, premenopausal females (*n* = 40; age range 25–44; NCT03848715), and older, postmenopausal females (*n* = 53; age range 60–80; NCT03256760). Men were not included because the study was designed to examine affective sensitivity to inflammation across female reproductive stages (i.e., premenopausal and postmenopausal). Sample recruitment methods were identical for the two groups, as previously described [37]. Eligibility criteria were similar for the two groups with the exception of age (younger group, range 25–44; older group, range 60–80) and are detailed in the study protocol [37]. Briefly, exclusion criteria included current psychiatric illness; active medical disorders; cognitive impairment; current use of antidepressants or medications known to influence the immune system; smoking; BMI > 35 kg/m^2^. Supplementary fig. S1 presents the CONSORT diagram.

### Trial procedures

During a baseline eligibility visit, participants provided written informed consent and completed self-report questionnaires and baseline behavioral reward tasks. Menopausal status for the age groups was confirmed by self-report during a medical history interview; those in the older group self-identified as postmenopausal and were at least four years past their last menstrual cycle. Those in the younger group self-identified as premenopausal, did not report current vasomotor symptoms (i.e., hot flushes and night sweats), and provided data on their last menstrual cycle, typical cycle length, and hormonal contraception use. Following eligibility assessment, participants were randomly assigned to receive either an infusion of low-dose endotoxin (0.8 ng/kg body weight) or placebo in a 1:1 ratio. Endotoxin challenge is a well-validated method for transiently inducing peripheral and central nervous system inflammation [38–40]. The experimental protocol was performed in the UCLA Clinical and Translational Science Institute (CTSI), as described in the published protocol [37]. Immediately before infusion (T0) and at hourly intervals post-infusion (T1-T9), participants completed self-report measures, provided blood samples, and had vital signs monitored. Behavioral reward tasks were readministered starting 2.5 h post-infusion, roughly corresponding with the overall peak of the inflammatory response to endotoxin observed in previous studies [10].

Randomization was stratified by age and performed using a computer-generated random number sequence by an independent researcher. To ensure allocation concealment, a secure, encrypted email was sent to the UCLA CTSI pharmacy. Investigators, outcome assessors, and participants were blind to allocation condition, with no instances of unblinding. Due to a national hold on endotoxin (May, 2021-October, 2022), the final allocation numbers were imbalanced, with more participants allocated to the placebo condition.

### Outcomes

The primary outcome was reward responsivity, including the domains of motivation, sensitivity, and learning. Reward motivation and reward sensitivity were assessed with the Effort Expenditure for Rewards Task (EEfRT). The EEfRT [41] is a computerized task that assesses effort-based decision-making for monetary reward. Briefly, participants are presented with a series of trials in which they choose between an easy, low-effort trial (worth a low reward amount of $1.00 and requiring 30 button presses) and a hard, high-effort trial (worth higher reward amounts ranging from $1.24-$4.30 and requiring 100 button presses). The EEfRT was shortened from 20–10 min as has been done previously [33–35]. Motivation for reward on the EEfRT was operationalized by willingness to exert effort for monetary reward; i.e., the selection of hard vs. easy trials. Sensitivity to reward was operationalized by the association between changes in monetary reward magnitude (ranging $1.24-$4.30) and changes in likelihood of selecting hard vs. easy trials [33–35]. Reward learning was assessed with the Probabilistic Reward Task (PRT). The PRT is a 15 min computerized task derived from signal detection theory which uses an asymmetric (3:1) pseudo-randomized reinforcement schedule to induce an implicit response bias towards one of two ambiguous stimuli [35, 42–44]. The total response bias was calculated across 200 trials at each administration of the task and was the index of reward learning. EEfRT and PRT task details are provided in supplementary material.

The secondary outcome was depressed mood scores. On the day of the experimental session, participants completed 10 items from the Profile of Mood States (POMS) Depressive subscale (unhappy, sad, blue, hopeless, discouraged, miserable, helpless, worthless, lonely, gloomy) at each timepoint (T0-T9). Items were rated on a 10-point scale (0 = not at all; 10 = extremely) and summed at each timepoint.

Other outcomes assessed were physical sickness symptoms, fatigue symptoms, and inflammation. Physical sickness symptoms at each timepoint (T0-T9) were a sum scale of the following items: shivering, nausea, breathing difficulties, muscle pain, joint pain, back pain, abdominal pain. Items were rated 0 (no symptoms) to 4 (very severe symptoms). Severity of fatigue was assessed as a single item on the same scale. Change scores over the first two hours were created for physical sickness symptoms and fatigue (T2-T0 symptoms) and used as covariates to ensure any effects of endotoxin were not attributable to changes in sickness or fatigue; sensitivity analyses using T1-T0 change scores yielded similar results.

As with our prior work [29, 45], circulating concentrations of the pro-inflammatory cytokines interleukin-6 (IL-6) and tumor necrosis factor-α (TNF-α) were assessed at T0 and hourly post-infusion as a measure of the inflammatory response to endotoxin or placebo. Plasma levels were quantified using a Meso Scale Discovery (MSD) multiple assay, as previously described [46]. Lower limits of detection were 0.2 pg/mL for IL-6 and 0.1 pg/mL for TNF-α; samples with concentrations below these limits were entered as 0.1 pg/mL (*n* = 3 IL-6 samples in the older group). Intra- and inter-assay precision of all tests was less than 9.8%.

### Adverse events

Adverse events were actively monitored with repeated assessment of vital signs, as well as occurrence of any severe sickness symptoms that impaired functioning and/or led to protocol non-completion. No adverse events were reported.

### Sample size

Prior endotoxin studies using behavioral tasks have found moderate to large effects of endotoxin on motivation [31] and, in female adults, depressed mood (*f* = 0.57) [13]. Assuming a moderate effect (*f* = 0.2) and correlation of 0.5 among repeated measures, a total sample size of 93 achieves >89% power for a within-between interaction (α = 0.05).

### Statistical analysis

Analyses were conducted using Stata version 13.1. Independent samples t-tests were used to test for baseline or T0 differences as a function of condition assignment and age group. Level of significance was set at *p* < 0.05 and hypothesis tests were two-sided. Adjusted models included change in physical sickness symptoms, change in fatigue symptoms, and T0 POMS depressed mood scores. Models with inflammatory markers included the additional covariates of BMI and race, as previously recommended [47].

#### EEfRT

Details on data reduction are provided in supplementary material. Decision-making behavior on the EEfRT was evaluated via generalized estimating equations (GEE) with a binary logistic model and independent working correlation structure. The working correlation structure was determined with the STATA program QIC (quasi-likelihood under the independence model criterion) [48]. Condition and age group (younger vs. older) were between-subject predictors, and time (baseline vs. post-infusion) was a within-subject predictor. The dependent variable was coded as 0 (low-effort/low-reward choice; easy) and 1 (high-effort/high-reward choice; hard). Three task-specific variables were included as continuous time-varying covariates: reward magnitude (range $1.24-$4.30), probability, and trial number (to control for fatigue effects). A condition by time by age group interaction term was the predictor of interest for reward motivation; for reward sensitivity, the predictor was a four-way interaction term between condition, time, age group, and reward magnitude.

#### PRT

Details on data reduction and cleaning using established inclusion criteria for evaluable data (e.g., valid number of trials) are provided in supplementary material. Mixed linear models were used to evaluate PRT response bias, with the predictor of interest a condition by time (baseline vs. post-infusion) by age group interaction term.

#### Depressed mood scores

Depressed mood was evaluated using mixed linear models with the predictor of interest a condition by time (hourly, starting at T0 through T9, 9 h post-infusion) by age group interaction term. POMS scores at each timepoint were natural log-transformed prior to analyses due to skewed distributions. Given a significant age group difference at T0, T0 POMS scores were included in the model (Supplementary Table S1).

#### Inflammation

IL-6 and TNF-α values were natural log-transformed prior to analyses due to skewed distributions at each timepoint. As with the POMS depressed mood analysis, mixed linear models were used with the predictor a condition by time by age group interaction. Given a significant age group difference at T0, T0 IL-6 and TNF-α values were included in the models. To probe the mechanistic role of inflammatory markers in endotoxin-induced changes in depressed mood and reward responsivity as a function of age group, we performed the analyses described above within the endotoxin condition, using change score variables for IL-6 and TNF-α in place of the condition variable. For the depressed mood outcome, change scores were calculated as T1-T0 levels, which temporally precedes the typical onset of depressed mood observed in previous studies [10, 11]. For the primary reward outcomes, change scores were calculated as T2-T0 levels, which temporally precedes the administration of reward tasks at 2.5 h post-infusion.

## Results

### Participant characteristics

Participants were 40 younger (*M*_*age*_ = 33.20 years) and 53 older (*M*_*age*_ = 66.64 years) female community-dwelling adults who were of normal BMI (*M* = 24.45, *SD* = 3.70) and identified as Asian (16%), Black or African American (12%), Other (12%), or White (60%). Depressive symptoms at the baseline visit on the Beck Depression Inventory-II were low (*M* = 1.72, *SD* = 2.44). The age groups did not differ on these characteristics (Supplementary Table S1). POMS depressed mood scores at T0 did differ, *t*(91) = 2.45, *p* = 0.02, with younger females reporting higher levels (*M* = 3.51, *SD* = 8.45) as compared to older females (*M* = 0.65, *SD* = 1.01).

There were age group differences in T0 levels of IL-6 and TNF-α, with older females showing significantly higher levels than younger females, as expected due to age differences [16] (*p*’s < 0.001; see Supplementary Table S1). There were no T0 age group differences in sickness symptoms or fatigue severity (*p*’s > 0.70), and no T0 condition differences in any other characteristic (*p*’s > 0.13). Seventeen younger participants (n = 13 placebo; n = 4 endotoxin) reported hormonal contraceptive use. Sensitivity analyses indicated that all results were slightly more robust but largely unchanged when excluding these participants. Therefore, all younger participants were included in the current report.

### Inflammatory response to endotoxin challenge as a function of age

**Fig. 1:**
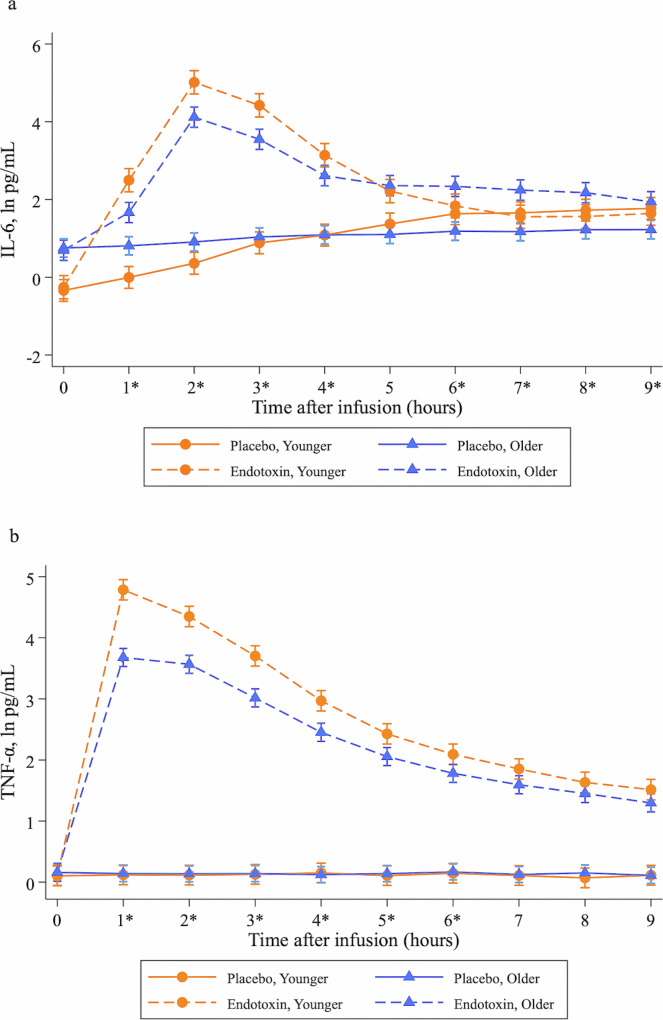
Changes in circulating levels of inflammatory markers. Changes over time in the endotoxin (dotted line) and placebo (solid line) conditions, stratified by group (circle for younger; triangle for older) in natural log-transformed plasma levels of (**a**) interleukin-6 (IL-6) and (**b**) tumor necrosis factor-α (TNF-α). Inflammatory markers were assessed at T0 (pre-infusion) and then hourly for 9 h post-infusion. Asterisks indicate significant condition by group interactions (*p* < 0.05) at individual timepoints, controlling for T0 IL-6 or TNF-α, using linear mixed models. Results are presented with 95% confidence intervals.

Linear mixed models showed that endotoxin, as compared to placebo, induced increases in IL-6 (condition x time effect: $\chi$^2^(9) = 889.91, *p* < 0.001) and TNF-α (condition x time effect: $\chi$^2^(9) = 5360.18, *p* < 0.001), which was moderated by age (condition x time x group effect: IL-6: ($\chi$^2^(9) = 239.79, *p* < 0.001); TNF-α: ($\chi$^2^(9) = 118.99, *p* < 0.001)). As shown in Fig. 1a, younger females showed a more robust increase in IL-6 in response to endotoxin vs. placebo as compared to older females, with overall peak IL-6 levels observed in both groups at T2. In the three-way interaction, the peak difference in IL-6 change between younger and older females occurred at 1 h post-injection (*b* = −1.50, *SE* = 0.30, *p* < 0.001; 95% CI [−2.08, −0.92]). While IL-6 returned to baseline at 6 h post-injection in the younger group, it remained elevated up to 9 h post-injection in the older group (all *p*’s < 0.002). For TNF-α, the younger group also showed a more robust increase, with both the overall peak levels and the peak difference as a function of the three-way interaction occurring at 1 h post-injection (*b* = −1.12, *SE* = 0.13, *p* < 0.001; 95% CI[−1.37, −0.87]; see Fig. 1b). Results were similar in analyses adjusting for BMI, race, and T0 POMS depressed mood, and the same pattern of findings was evident when testing IL-6 and TNF-α change scores in multiple regression models (see Supplementary Material).

### Primary outcome: reward response to endotoxin challenge as a function of age

#### EEfRT reward sensitivity response

**Fig. 2:**
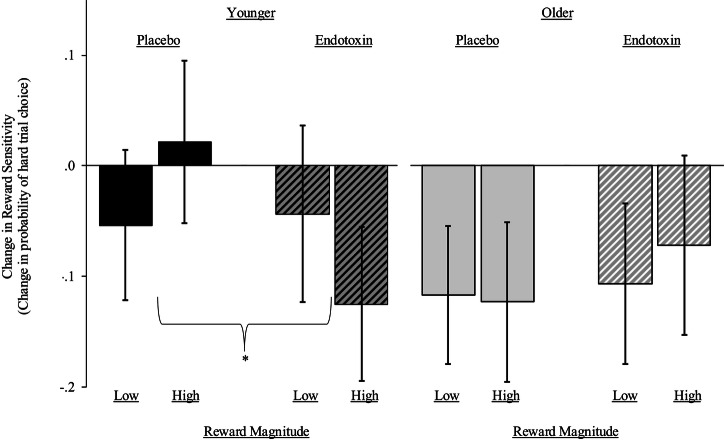
Changes in reward sensitivity. Changes over time in reward sensitivity in the endotoxin and placebo conditions, stratified by group (younger in black, older in grey), as tested by the probability of selecting hard trials when reward magnitude is high vs. low. Reward magnitude on the x-axis is divided into high ($3.02-$4.30) and low ($1.24-$2.66) monetary categories. The y-axis shows the difference from baseline to 2.5 h post-infusion in the probability of choosing hard trials on the EEfRT. The asterisk indicates a significant condition by time by reward magnitude interaction in the younger group (*p* = 0.03) with no differences in the older group (*p* = 0.57). Results are presented with 95% confidence intervals.

It was hypothesized that endotoxin would decrease sensitivity to monetary reward, as represented by a weakened association between reward magnitude (amount of money offered for hard trials) and the selection of hard vs. easy trials, to a greater degree in younger vs. older female adults. Consistent with this hypothesis, the condition by time by reward magnitude by age interaction was significant (*b* = 0.13, *SE* = 0.05, *p* = 0.01; 95% CI[0.03, 0.23]) and the condition by time by reward magnitude interaction was not significant (*p* = 0.32). To aid in interpretation and visualization, we conducted follow-up analyses treating reward magnitude as a categorical (low vs. high) variable; the four-way interaction remained significant ($\chi$^2^(1) = 5.84, *p* = 0.02). As shown in Fig. 2, endotoxin decreased reward sensitivity in younger females, as evidenced by the decreased probability of choosing hard trials when the reward magnitude was high (*b* = −0.15, *SE* = 0.05, *p* = 0.004; 95% CI[−0.25, −0.05]) but not low (*p* = 0.85). There were no differences in the older group (*p*’s >0.42). Results were similar in adjusted analyses (Supplementary Table S2). Changes in inflammatory markers within the endotoxin condition did not predict change in reward sensitivity in younger vs. older female adults (*p’s* > 0.18).

#### EEfRT reward motivation response

The acute inflammatory challenge vs. placebo was hypothesized to decrease reward motivation to a greater degree in younger vs. older female adults. However, neither the condition by time interaction (*p* = 0.51) nor the condition by time by age interaction was significant (*b* = 0.38, *SE* = 0.22; *p* = 0.09; 95% CI[−0.06, 0.81]; see Supplementary fig. S2). Adjusted analyses were similar (Supplementary Table S3).

**Fig. 3:**
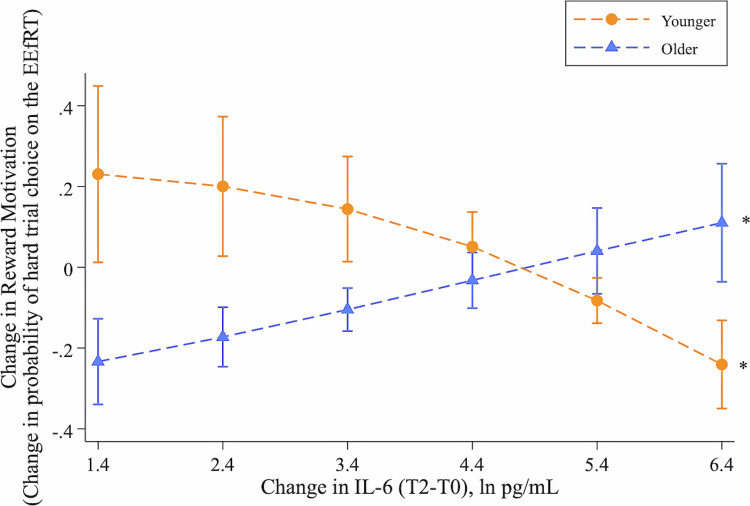
Association between change in reward motivation and change in IL-6 following acute inflammatory challenge in younger (circles) and older (triangles) female adults. The x-axis shows increments of change in circulating levels of IL-6 at two hours following endotoxin infusion (calculated as the difference in natural log-transformed IL-6 values from T0 to T2). The y-axis shows increments of the change in reward motivation, calculated as the difference in the probability of hard trial choice on the Effort Expenditure for Rewards Task (EEfRT) from baseline to 2.5 h post-infusion. Asterisks indicate that increased IL-6 is associated with decreased reward motivation in the younger group and increased reward motivation in the older group using generalized estimating equations (*p’s <0*.05). Results are presented with 95% confidence intervals.

Within the endotoxin condition, the interaction between change in IL-6 (T2-T0), time, and age predicted reward motivation (*b* = 0.93, *SE* = 0.22; *p* < 0.0001; 95% CI[0.50, 1.37]), such that greater increases in IL-6 were associated with decreased motivation, as measured by decreased selection of hard trial choice, among younger female adults ($\chi$^2^(1) = 12.28, *p* = 0.0005; see Fig. 3). By contrast, greater increases in IL-6 were associated with increased motivation in older female adults ($\chi$^2^(1) = 8.25, *p* = 0.004). The interaction between change in TNF-α, time, and age was not significant in adjusted models (*b* = 0.34, *SE* = 0.19; *p* = 0.07; 95% CI[−0.02, 0.71]).

#### PRT reward learning response

We next evaluated whether endotoxin vs. placebo decreased reward learning on the PRT to a greater degree in younger vs. older female adults. There was a significant condition by time interaction (*b* = −0.25, *SE* = 0.12, *p* = 0.04; 95% CI[−0.48, −0.01]; see Supplementary fig. S3) that was not moderated by age (*p* = 0.51). Follow-up analyses showed a decrease over time in reward learning in the endotoxin condition (*b* = −0.30, *SE* = 0.09, *p* = 0.0008; 95% CI[−0.47, −0.12]) but not the placebo condition (*p* = 0.51). Results were similar in adjusted analyses (Supplementary Table S4). Increases in IL-6 or TNF-α within the endotoxin condition were not associated with change in PRT reward learning, controlling for age group (*p’s* > 0.53).

### Secondary outcome: depressed mood response to endotoxin challenge as a function of age

**Fig. 4:**
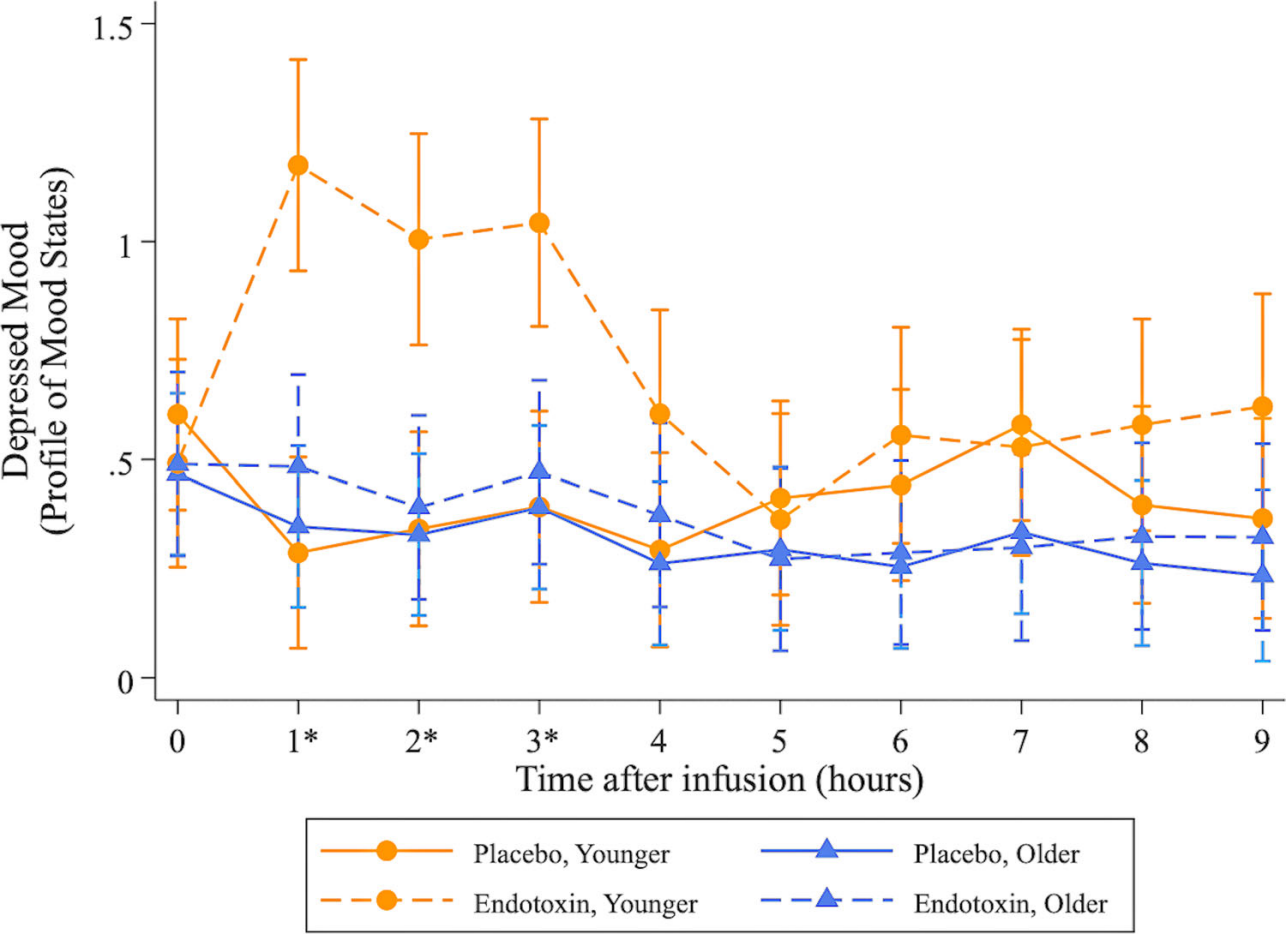
Changes in depressed mood. Changes over time in the endotoxin (dotted line) and placebo (solid line) conditions, stratified by group (circle for younger; triangle for older) in depressed mood as measured by items from the Profile of Mood States Depressive subscale (natural log-transformed on the y-axis). Depressed mood was assessed at T0 (pre-infusion) and then hourly for 9 h post-infusion. Asterisks indicate significant condition by group interactions (*p* < 0.05) at individual timepoints controlling for T0 depressed mood using linear mixed models. Results are presented with 95% confidence intervals.

Linear mixed models showed that endotoxin, as compared to placebo, induced increases in depressed mood (condition x time effect, $\chi$^2^(9) = 37.76, p < 0.0001), and this was moderated by age (condition x time x group effect, $\chi$^2^(9) = 29.58, *p* = 0.0005). As shown in Fig. 4, the acute inflammatory challenge increased depressed mood in younger ($\chi$^2^(9) = 66.49, *p* < 0.0001) but not older ($\chi$^2^(9) = 2.27, *p* = 0.99) female adults. This effect peaked at 1 h post-infusion (*b* = −0.89, *SE* = 0.23, *p* = 0.0002; 95% CI[−1.34, −0.43]) and lasted through 3 h post-infusion, with similar effects evident when adjusting for changes in sickness symptoms and fatigue. Raw scores on the POMS as a function of age group and condition assignment are provided in Supplementary Table S5.

To explore the role of inflammatory markers in inducing depressed mood, we then tested a mixed model with a change in IL-6 (T1-T0) by time by age interaction, predicting depressed mood within the endotoxin condition. This interaction was significant ($\chi$^2^(9) = 18.58, *p* = 0.03; see Supplementary fig. S4), and follow-up analyses indicated that increases in IL-6 were associated with increased depressed mood from T0 to T2 in younger females (*b* = 0.39, *SE* = 0.17, *p* = 0.03; 95% CI[0.05, 0.72]), but not older females (*p* = 0.72). A similar pattern did not reach significance for change in TNF-α ($\chi$^2^(9) = 13.49, *p* = 0.14). Adjusted analyses yielded similar results.

## Discussion

Depression prevalence is nearly twice as high in females compared to males [49], with a markedly higher prevalence in younger as compared to older female adults [5, 49]. This study used an experimental model of acute inflammation to test heightened affective response to inflammation as a potential mechanism of differential vulnerability between younger and older females. While endotoxin administration increased pro-inflammatory cytokines in both the younger and older groups, only younger females exposed to LPS exhibited increased scores for depressed mood, reduced sensitivity to reward, and reduced motivation in association with greater increases in IL-6. Contrary to hypotheses, both groups showed endotoxin-induced deficits in reward learning.

These findings indicate that younger females are more affectively vulnerable to inflammatory activation than older females. Aging encompasses multiple factors that may provide protective benefits. These include decreased interoception [50], which is linked to less physiological arousal to emotional stimuli [51], improved emotion regulation [52], or, as observed in the current study, an attenuated inflammatory response to the acute challenge in older females compared to younger females. In addition, the reduced fluctuations in sex steroids observed during postmenopause, compared to premenopause, may also play a critical role. Sex steroid fluctuations induce neurochemical alterations relevant for depressive behavior [53, 54] and are linked to higher depression risk (e.g., during major hormonal transitions like puberty or perimenopause) [55]. Moreover, as compared to age-matched males, younger female adults show evidence of greater endotoxin-induced activation of the cAMP response element binding protein (CREB) signaling pathway in peripheral blood mononuclear cells [56], which could be driven by differences in prostaglandin production [57]. Prostaglandins mediate endotoxin-induced depressive behaviors in animal models [58, 59] and regulate female reproductive health (e.g., menstruation, ovulation, labor induction in pregnancy). Thus, this work supports further research directly testing the intersection of hormone fluctuations and inflammatory signaling to better understand disparities in depression prevalence. These findings also highlight the importance of extending this work to the perimenopausal period, which is characterized by both dynamic hormonal fluctuations and heightened risk for depressive symptoms.

Reward motivation is consistently linked to inflammation [23], but this study found no effect of endotoxin versus placebo on motivation. However, increases in inflammatory markers correlated with changes in reward motivation, suggesting that shifts in motivation depend on the magnitude of the inflammatory response. Intriguingly, increased IL-6 predicted decreased reward motivation in the younger group, but increased reward motivation in the older group. This suggests older females exposed to inflammation may have a reduced risk of motivational deficits. Whether this is a mechanism contributing to lower vulnerability to depression from acute inflammatory events in this group merits further study.

Reward sensitivity has weaker links to inflammation in the literature [23], and our work found endotoxin-induced deficits in sensitivity only in the younger group. This blunted sensitivity was not associated with changes in inflammatory markers, which may indicate that shifts in reward sensitivity are less dependent on the magnitude of the inflammatory response and instead reflect individual differences in vulnerability to similar levels of inflammation. Alternatively, the endotoxin challenge may have induced central nervous system changes, such as microglial activation [60], that could more directly alter reward sensitivity but were not measured in this study. Signaling from peripheral inflammation, as well as central inflammation, could differentially alter the neural regions underlying reward sensitivity, such as the orbitofrontal cortex (involved in the evaluation and integration of monetary reward outcomes) versus the mesolimbic pathways that govern reward motivation [23].

Both younger and older females exhibited endotoxin-induced deficits in reward-related learning, as tested by the PRT. This finding may result from the implicit nature of the PRT, as any advantage associated with improved emotion regulation in older age [52] could be diminished compared to self-reported mood or performance on explicit tasks like the EEfRT. Older age is also associated with changes in dopaminergic function [61], potentially increasing vulnerability to learning tasks reliant on striatal dopamine function [62]. This suggests a vulnerability in older age that may require targeted treatment.

Strengths of this study include the rigorous experimental design, use of the endotoxin model to reliably induce inflammation, and use of behavioral reward tasks to capture multiple reward domains. Characterizing the effects of inflammation on specific dimensions of reward behavior is relevant to anhedonia and has important implications for the targeted treatment of depression. One limitation is the sole use of monetary rewards. Inflammation has differential effects on social versus monetary reward [63], and aging is associated with socioemotional shifts, including greater prioritization of close social connections [52]. Thus, it will be important to test whether the differential vulnerability observed in this report extends to social reward responses. Additionally, the measure of reward sensitivity in this study involves the cognitive evaluation of monetary reward magnitude. Future work should evaluate the initial affective hedonic response to capture more automatic processes. Our assessment of reward learning is also limited in its specificity, as performance on the PRT encompasses both learning and sensitivity parameters [35, 64]. Finally, this study demonstrates that acute inflammation induces transient increases in depressed mood scores in non-depressed individuals, and the relevance of these results for clinical depression requires further study. Despite these limitations, this study provides the first experimental evidence that younger female adults are more vulnerable to both inflammation-induced increases in scores for depressed mood and inflammation-induced anhedonic behavior than are older female adults. Although these findings require replication, they suggest a plausible interplay between hormonal fluctuations and inflammatory signaling that may be relevant for developing targeted prevention and treatment of depression in younger female adults.

## Supplementary information

Supplementary Material

## Data Availability

Data will be made available on request to CCB and MRI (ccboyle@ucla.edu; mirwin1@ucla.edu).
