# Supplementary material for: Inflammation-induced depressed mood and reward responsivity as a function of age in female adults: a randomized controlled trial of endotoxin
Source: Transl Psychiatry. 2025 Nov 26;16:6. doi: 10.1038/s41398-025-03752-2 (PMC12783272; doi:10.1038/s41398-025-03752-2)
Supplement: Supplementary file 1 — Supplementary Material [file 41398_2025_3752_MOESM1_ESM.docx]

**Inflammation-induced depressed mood and reward responsivity as a function of age in female adults: A randomized controlled trial of endotoxin**

Chloe C. Boyle, PhD; Joshua H. Cho, MD, PhD; Naomi I. Eisenberger, PhD; Richard Olmstead, PhD; Nina Sadeghi, BS, Daisy Castillo, BS, Michael R. Irwin, MD

**Supplementary material**

1. Title and Index.
2. Table S1. Demographic and psychosocial characteristics of the younger and older female adults.
3. Table S2. Reward sensitivity is reduced by acute inflammatory challenge in younger, but not older, female adults.
4. Table S3. Reward motivation is not altered by acute inflammatory challenge in younger and older female adults.
5. Table S4. Reward learning on the Probabilistic Reward Task (PRT) is reduced by acute inflammatory challenge in younger and older female adults.
6. Table S5. Means and standard deviations for raw POMS Depressed Mood scores as a function of age group and condition assignment.
7. Figure S1. CONSORT Diagram.
8. Figure S2. Change in reward motivation in the endotoxin vs. placebo condition (split by age group).
9. Figure S3. Change in reward learning in the endotoxin vs. placebo condition.
10. Figure S4. Association between change in IL-6 and change in depressed mood as a function of age group within the endotoxin condition.
11. Study Recruitment Details.
12. Study Procedures Details.
13. EEfRT and PRT Data Reduction and Cleaning.
14. PRT Task Description and Response Bias Equation.
15. Results from regression models testing the inflammatory response to endotoxin challenge as a function of age group using change scores.
16. References.

**Table S1.** Demographic and psychosocial characteristics of the younger and older female adult participants.

|  | Younger  (*n* = 40) | Older  (*n* = 53) | Tests for Group Differences^a^ |
| --- | --- | --- | --- |
| Variable |  |  |  |
| Age, mean years (*SD*) | 33.2 (5.99) | 66.64 (5.45) | *t*(91) = -28.07, *p* < .001 |
| BMI, mean (*SD*) | 25.18 (4.36) | 23.89 (3.03) | *t*(90) = 1.67, *p* = .10 |
| Race, *n* (%) |  |  | $X$^2^ = 6.53, *p* = .09 |
| Asian | 7 (17.50%) | 8 (15.09%) |  |
| Black/African American | 6 (15%) | 5 (9.43%) |  |
| Other | 8 (20%) | 3 (5.66%) |  |
| White | 19 (47.50%) | 37 (69.81%) |  |
| Ethnicity, *n* (%) |  |  | $X$^2^ = 2.12, *p* = .15 |
| Hispanic/Latinx | 8 (20%) | 5 (9.43%) |  |
| Education, Bachelor’s degree and higher, n (%) | 31 (81.58%) | 40 (75.47%) | $X$^2^ = 0.48, *p* = .49 |
| Depressive symptoms, mean (*SD*) | 1.65 (2.62) | 1.78 (2.33) | *t*(91) = 0.25, *p* = .81 |
| Anxiety symptoms, mean (*SD*) | 2.10 (2.89) | 1.38 (2.02) | *t*(91) = 1.40, *p* = .17 |
| Sleep disturbance symptoms, mean (*SD*) | 1.28 (1.89) | 1.06 (1.29) | *t*(91) = 0.66, *p* = .51 |
| T0 depressed mood, mean (*SD*) | 3.51 (8.45) | 0.64 (1.01) | *t*(91) = 2.45, *p* = .02 |
| T0 fatigue, mean (*SD*) | .45 (.81) | .40 (.60) | *t*(91) = 0.37, *p* = .72 |
| T0 physical sickness symptoms, mean (*SD*) | .20 (.41) | .21 (.53) | *t*(91) = -0.08, *p* = .94 |
| T0 IL-6 pg/ml, mean (SD) | .4 (.3) | 5.2 (2.9) | *t*(91) = -10.35, *p* <.001 |
| T0 TNF-α pg/ml, mean (SD) | .8 (.2) | 1.8 (0.9) | *t*(91) = -6.98, *p* <.001 |

*Note*. Demographic variables and behavioral symptom variables were assessed at the baseline screening visit; all other variables are reported from T0, immediately before infusion on the day of the experimental session. Depressive symptoms were assessed with the Beck Depression Inventory-II (higher values indicate greater severity of symptoms). T0 depressed mood was assessed with items from the Profile of Mood States (POMS) questionnaire. Raw values of IL-6 and TNF-α are reported for means and standard deviations (SD).

^a^Group differences for each variable were tested with independent samples t-tests or chi-square tests as appropriate.

**Table S2.** Reward sensitivity is reduced by acute inflammatory challenge in younger, but not older, female adults.

| Variable | *b* | *SE* | *Z* | *p* |
| --- | --- | --- | --- | --- |
| Time | -0.598 | 0.249 | -2.40 | 0.016 |
| Condition | 0.640 | 0.264 | 2.42 | 0.015 |
| Condition X time | 0.479 | 0.371 | 1.29 | 0.197 |
| Group | 0.315 | 0.236 | 1.33 | 0.182 |
| Time X group | 0.027 | 0.325 | 0.08 | 0.934 |
| Condition X group | -0.372 | 0.344 | -1.08 | 0.280 |
| Condition X time X group | -0.601 | 0.488 | -1.23 | 0.218 |
| Reward magnitude | 0.149 | 0.018 | 8.46 | <. 001 |
| Time X reward magnitude | 0.053 | 0.026 | 2.06 | 0.040 |
| Condition X reward magnitude | 0.055 | 0.029 | 1.87 | 0.061 |
| Condition X time X reward magnitude | -0.100 | 0.041 | -2.45 | 0.014 |
| Group X reward magnitude | -0.056 | 0.023 | -2.40 | 0.016 |
| Time X Group X reward magnitude | -0.042 | 0.034 | -1.24 | 0.216 |
| Condition X group X reward magnitude | -0.061 | 0.038 | -1.61 | 0.107 |
| Condition X time X group X reward magnitude | 0.126 | 0.052 | 2.41 | 0.016 |
| Probability | 0.491 | 0.037 | 13.40 | <. 001 |
| Trial number | -0.044 | 0.003 | -14.7 | <. 001 |
| Change in fatigue severity | -0.022 | 0.041 | -0.55 | 0.581 |
| Change in physical sickness symptoms | -0.195 | 0.047 | -4.11 | 0.821 |
| T0 depressed mood | -0.077 | 0.038 | -2.05 | 0.408 |
| Intercept | -0.709 | 0.184 | -3.86 | .0001 |

*Note*. Age group is coded as younger (0) relative to older (1). Reward magnitude refers to the monetary value for each hard (i.e., high effort/high reward) trial, ranging from $1.24-$4.30. Probability for each trial was low (12%) medium (50%) or high (88%). Baseline depressive mood was assessed with items from the Profile of Mood States questionnaire.

**Table S3.** Reward motivation is not altered by acute inflammatory challenge in younger and older female adults.

| Variable | *b* | *SE* | *Z* | *p* |
| --- | --- | --- | --- | --- |
| Time | -0.140 | 0.113 | -1.24 | 0.215 |
| Condition | 1.023 | 0.139 | 7.34 | <. 001 |
| Condition X time | -0.303 | 0.179 | -1.69 | 0.090 |
| Group | -0.155 | 0.133 | -1.17 | 0.243 |
| Time X group | -0.350 | 0.152 | -2.3 | 0.022 |
| Condition X group | -0.802 | 0.174 | -4.6 | <. 001 |
| Condition X time X group | 0.404 | 0.237 | 1.71 | 0.088 |
| Probability | 0.487 | 0.036 | 13.36 | <. 001 |
| Reward magnitude | 0.133 | 0.006 | 20.7 | <. 001 |
| Trial number | -0.043 | 0.003 | -14.78 | <. 001 |
| Change in fatigue severity | -0.027 | 0.040 | -0.66 | 0.507 |
| Change in physical sickness symptoms | -0.192 | 0.047 | -4.08 | <. 001 |
| T0 depressed mood | -0.077 | 0.037 | -2.06 | 0.040 |
| Intercept | -0.571 | 0.121 | -4.72 | <. 001 |

*Note*. Age group is coded as younger (0) relative to older (1). Reward magnitude refers to the monetary value for each hard (i.e., high effort/high reward) trial, ranging from $1.24-$4.30. Probability for each trial was low (12%) medium (50%) or high (88%). T0 depressed mood was assessed with items from the Profile of Mood States questionnaire.

**Table S4.** Reward learning on the Probabilistic Reward Task is reduced by acute inflammatory challenge in younger and older female adults.

| Variable | *b* | *SE* | *Z* | *p* |
| --- | --- | --- | --- | --- |
| Time | -0.043 | 0.079 | -0.55 | 0.5851 |
| Condition | 0.044 | 0.092 | 0.49 | 0.626 |
| Condition X time | -0.249 | 0.118 | -2.11 | 0.035 |
| Group | 0.052 | 0.094 | 0.56 | 0.577 |
| Change in fatigue severity | 0.067 | 0.039 | 1.72 | 0.085 |
| Change in physical sickness symptoms | 0.035 | 0.045 | 0.79 | 0.431 |
| T0 depressed mood | -0.001 | 0.042 | -0.01 | 0.991 |
| Intercept | 0.372 | 0.082 | 4.54 | <. 001 |

*Note*. Age group is coded as younger (0) relative to older (1). Baseline depressive mood was assessed with items from the Profile of Mood States questionnaire.

**Table S5.** Means and standard deviations for raw POMS Depressed Mood scores as a function of age group and condition assignment

| Time | Younger Age Group | | Older Age Group | |
| --- | --- | --- | --- | --- |
|  | Placebo (M, SD) | Endotoxin (M, SD) | Placebo (M, SD) | Endotoxin (M, SD) |
| 0 | 3.95 (6.45) | 2.97 (10.57) | 0.57 (1.00) | 0.74 (1.03) |
| 1 | 2.96 (6.27) | 7.07 (11.26) | 0.33 (0.71) | 0.93 (1.81) |
| 2 | 2.86 (4.90) | 6.78 (16.16) | 0.28 (0.58) | 0.55 (0.85) |
| 3 | 3.57 (6.73) | 5.75 (11.52) | 0.32 (0.58) | 0.82 (1.36) |
| 4 | 4.29 (8.83) | 2.23 (4.86) | 0.16 (0.36) | 0.54 (1.02) |
| 5 | 4.45 (9.03) | 1.96 (5.82) | 0.23 (0.53) | 0.34 (0.71) |
| 6 | 4.25 (8.70) | 1.46 (3.80) | 0.16 (0.40) | 0.34 (0.64) |
| 7 | 5.47 (10.55) | 1.46 (3.80) | 0.33 (0.72) | 0.44 (1.01) |
| 8 | 3.76 (8.07) | 3.24 (8.00) | 0.16 (0.41) | 0.45 (0.81) |
| 9 | 3.16 (7.06) | 2.74 (5.50) | 0.09 (0.36) | 0.54 (1.20) |

*Note*. Time is in hours relative to infusion (0= pre-infusion). M=Mean; SD=Standard Deviation; POMS=Profile of Mood States.

**Figure S1. CONSORT diagram**

Eligibility Screening/ Baseline (n = 455)

Ineligible at Screening/Baseline: **Total n=** **362**

- Baseline HR < 50: **28**
- Abnormal ECG: **36**
- Use of Exclusion Medication: **29**
- Substance Use: **2**
- Sleep Disorder (Diagnosed Sleep Apnea/ RLS/ Phase Shift): **26**
- Positive Berlin Sleep Apnea Quest.: **16**
- BMI > 35: **9**
- Current MDD or Bipolar Disorder: **17**
- Suicidal Ideation/ Hx of Suicide Attempt: **4**
- Active Medical Condition: **43**
- Cognitive Impairment: **1**
- Limited English Proficiency: **4**
- Problems with IV/no infusion: **1**
- Withdrew/ Declined: **39**
- Male: **54**
- Insomnia Diagnosis: **53**

**Enrollment**

40 Younger Participants

53 Older Participants

53 Older participants randomized

40 Younger participants randomized

**Allocation**

Randomized to Placebo n = 30

Randomized to Placebo n = 22

Randomized to Endotoxin n = 23

Randomized to Endotoxin n = 18

**Analysis**

Analyzed

Depressed Mood (n=23)

- Excluded from analyses (n=0)

EEfRT (n=23)

- Excluded from analyses (n=0)

PRT (n=23)

- Excluded from analyses (n=0)

Analyzed

Depressed Mood (n=22)

- Excluded from analyses (n=0)

EEfRT (n=22)

- Excluded from analyses (n=0)

PRT (n=20)

- Excluded from analyses (n=2); PRT data at both timepoints did not pass quality control assessments

Analyzed

Depressed Mood (n=18)

- Excluded from analyses (n=0)

EEfRT (n=18)

- Excluded from analyses (n=0)

PRT (n=16)

- Excluded from analyses (n=2); PRT data at both timepoints did not pass quality control assessments

Analyzed

Depressed Mood (n=30)

- Excluded from analyses (n=0)

EEfRT (n=30)

- Excluded from analyses (n=0)

PRT (n=29)

- Excluded from analyses (n=1); PRT data at both timepoints did not pass quality control assessments

**Figure S2.**

**
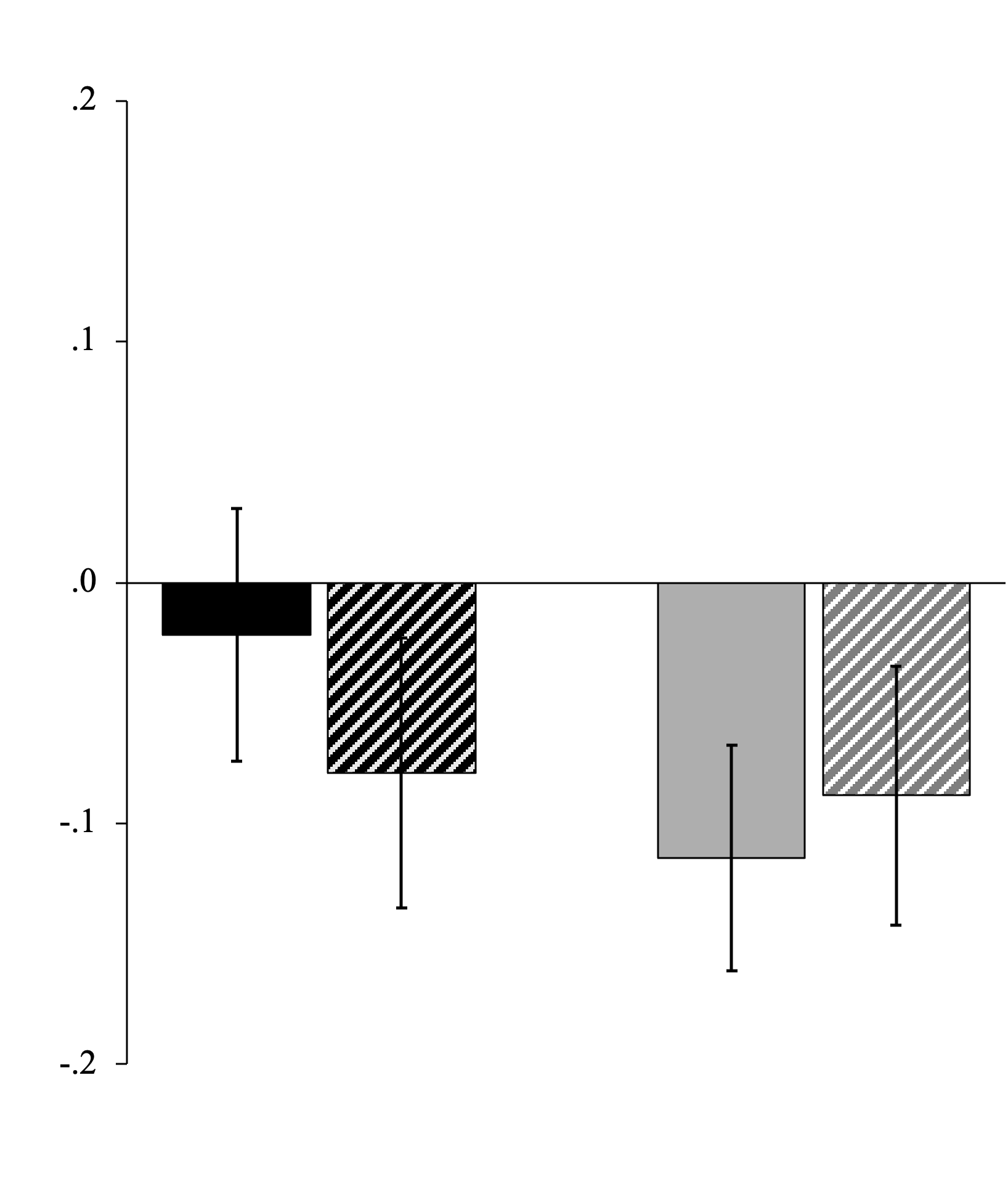
**

Older

Younger

Change in Reward Motivation

(Change in probability of hard trial choice on the EEfRT)

Placebo

Endotoxin

Endotoxin

Placebo

*****

*****

*****

**Figure S2.** Change in reward motivation in the endotoxin vs. placebo condition (split by age group). As noted in the main text, the overall 3-way interaction between condition, time, and age group was not significant (p=.088). Asterisks indicate marginal means that are significantly different from 0 (*p*’s <.005). The y axis shows change in the probability of hard trial choice on the Effort Expenditure for Rewards Task (EEfRT) from pre- to post-infusion. Results are depicted with 95% confidence intervals.

**Figure S3.**

Condition

Change in Reward Learning

(Change in response bias score on the PRT)

Placebo

Endotoxin

*****

**Figure S3.** Change in reward learning. Change over time in the endotoxin (striped bar) and placebo (black bar) conditions in reward learning, as tested by the response bias score on the Probabilistic Reward Task (PRT). The y-axis depicts change in the response bias score from baseline to post-infusion. The asterisk indicates a significant condition by time interaction (*p*<.05) using linear mixed models, with reward learning decreasing only in the endotoxin condition. Analyses control for age group; results are similar when excluding this variable. Results are presented with 95% confidence intervals.

**Figure S4.**

Depressed Mood

(Profile of Mood States Score)

*****

Time after infusion (hours)

Younger, IL-6 (mean)

Older, IL-6 (mean)

Younger, IL-6 (mean - 1SD)

Younger, IL-6 (mean + 1SD)

Older, IL-6 (mean - 1SD)

Older, IL-6 (mean + 1SD)

**Figure S4.** Association between change in IL-6 and change in depressed mood as a function of age group within the endotoxin condition. For descriptive purposes, change in depressed mood is shown separately for the younger (black lines) and older (grey lines) group and split by degree of change in IL-6 (change score calculated as T1-T0 levels). Change in IL-6 is depicted at 1 standard deviation (SD) below the mean change in IL-6 (dot), mean level of change in IL-6 (dash), and 1 SD above the mean change in IL-6 (short dash). Depressed mood was assessed at baseline (T0) and then every hour after infusion until 9-hours post-infusion. The asterisk indicates a significant association between change in IL-6 (T1-T0) and change in depressed mood at T2 in the younger, but not older, group. Results are depicted with 95% confidence intervals.

**Study Recruitment Details**

All older adult participants (age 60-80) were recruited from the Los Angeles community August 2017-November 2022 using the GENESYS Sampling Systems company, which provided contact information for households with at least one person aged 60 years or older within a 10-mile radius of UCLA. For the younger female adults, participants were recruited using the same methods from October 2019-July 2022 with the age range restricted to individuals 25-44. For the younger female adults, 68 individuals responded to recruitment efforts and completed eligibility screening by phone and in-person. Of these, 40 were eligible and enrolled in the study (n=18 endotoxin; n =22 placebo).

**Study Procedure Details**

During a baseline eligibility visit, participants provided written informed consent, vital signs, and an electrocardiogram. The baseline assessment also included administration of the SCID-DSM-5 by trained research staff and completion of self-report questionnaires and baseline behavioral tasks. Menopausal status was confirmed by self-report during a medical history interview. Within approximately two weeks, participants were scheduled to return to the UCLA Clinical and Translational Research Center (CTRC) to complete the experimental protocol. Participants arrived at 7:30AM and had their height, weight, and vitals taken by nursing staff. Ninety minutes later, they were randomly assigned to receive either an infusion of low dose endotoxin (0.8 ng/kg body weight) or placebo (same volume of 0.9% saline). A random allocation sequence was generated by an independent researcher using a computerized uniform random number generator and done in coordination with the UCLA Pharmacy for maintenance of drug blinding. A nurse, who was blind to condition, inserted a catheter with a heparin lock into the dominant forearm for blood draws and one into the non-dominant forearm for drug administration and a continuous saline flush. Participants completed self-report measures and provided blood samples at baseline (T0) and at hourly intervals post-infusion (T1-T9) for assessment of circulating levels of the pro-inflammatory cytokines interleukin-6 (IL-6) and tumor necrosis factor-α (TNF-α). Physical sickness symptoms, fatigue, and vital signs were assessed at each blood draw. Participants completed the behavioral reward tasks beginning at 2.5 hours post-infusion, roughly corresponding with the known peak of the inflammatory response to endotoxin (1). Participants were discharged from the CTRC at 6:00PM with physical and psychological symptoms returned to baseline and received $750 (younger) or $1000 (older) for their participation. The endotoxin for the current study (LOT 94332B1) was derived from *Escherichia Coli* and manufactured by LIST Biological Laboratories (E.coli O:113) under contract with the Clinical Center at NIH. It is GMP grade material suitable for Phase I trials in humans (BB-IND 12948 to M.R.I.). Endotoxin has been widely used to safely examine the effects of experimental inflammation in humans by our group and others (2–5) and mimics increases in circulating cytokines that are similar to what is reported in chronic low-grade inflammatory disorders (6,7).

**EEfRT and PRT Data Reduction and Cleaning**

**EEfRT Data Reduction.**

At baseline, participants chose between 18 and 48 trials (younger) or 12 and 51 trials (older); two older participants chose fewer than 3 trials at baseline and their baseline data only was treated as missing. At post-infusion, participants chose between 24 and 45 trials (younger) or 21 and 51 trials (older). Individual trials in which the participant did not choose between an easy or hard task were excluded (younger: 3.03% of all baseline trials; 1.72% of all post-infusion trials; older: 5.14% of all baseline trials: 3.95% of all post-infusion trials). Trials that the participant selected but completed fewer than 75% of the required button presses were excluded (younger: 1.76% at baseline; 2.59% at post-infusion; older: 2.57% at baseline; 2.84% at post-infusion).

**PRT Data Reduction and Cleaning.**

For analyses in the current study inclusion criteria for evaluable PRT data were: accuracy greater than 50%; ratio of rewards received greater than 2.4; at least 80% trials within valid range (150ms-2500ms); fewer than 16 outliers. Five participants (four from the younger group) were excluded from analyses (5.4% of the sample; n=3 placebo; n=2 endotoxin) for having data that did not meet established inclusion criteria for evaluable data at both timepoints. An additional 15 participants had missing data at one of the two timepoints (n=8 younger with 5 at baseline and 3 at post; n=7 older with 4 at baseline and 3 at post).

**Probabilistic Reward Task (PRT) Task Description and Response Bias Equation**


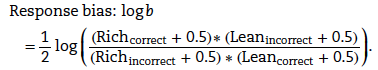
The PRT is a 15-minute computerized task that is derived from signal detection theory and assesses the development of an implicit response bias towards more frequently rewarded stimuli (Pizzagalli et al., 2005). Response bias is a total score on the task that encompasses both implicit *learning rate* and *sensitivity* to reward cues (e.g., Huys et al., 2013) and has been termed *reward responsiveness* (e.g., Bogdan & Pizzagalli, 2006). The response bias score is calculated with the formula below; Rich refers to the more frequently rewarded stimulus, and Lean refers to the less frequently rewarded stimulus.

Participants completed two 7 minute blocks of 100 trials each, with a 30-sec break between blocks. For each trial, participants were asked to identify which of two difficult-to-differentiate stimuli were presented. The stimuli were cartoon faces with one of two straight mouths (10mm short mouth versus 11mm long mouth). Each trial presented a fixation cross [750ms], a mouthless cartoon face [500ms], and then a face with a mouth [100ms]. Participants made their choice of mouths by pressing the ‘c’ or ‘m’ key (counterbalanced) and were then presented with either feedback *(“Correct! You won 20 cents!”)* or a blank screen [1750ms]. Both the long and short stimuli are presented equally often, but an asymmetric (3:1) pseudo-randomized reinforcement schedule is used to induce a response bias towards the more frequently rewarded stimuli across the 200 trials. The same stimulus was never presented more than three times consecutively, and if participants did not respond correctly to a trial scheduled for reward, reward feedback was delayed until the next correct identification of that stimulus. This differential reinforcement schedule reliably elicits a response bias towards the more frequently rewarded stimulus in healthy controls (e.g., Pizzagalli, 2005). Participants completed 10 practice trials to familiarize themselves with the task, and two versions of the PRT (counterbalanced across participants) were administered, one with mouth stimuli and one with nose stimuli (long and short were 5.31mm and 5.00mm, respectively). Participants received $10 for each administration of the task.

**Results from regression models testing the inflammatory response to endotoxin challenge as a function of age group using change scores**

Consistent with results from linear mixed models, regression models evaluating change in IL-6 and TNF-α using change scores indicated a more robust response to endotoxin versus placebo in the younger as compared to older female adults. For T1-T0 change scores, there were significant condition by age group interactions for both change in IL-6 (*b*=-1.50, *SE*=0.32, *p*<.001; 95% CI[-2.15, -0.86]) and change in TNF-α (*b*=-1.12, *SE*=0.28, *p*<.001; 95% CI[-1.68, -0.56]), such that increases in IL-6 and TNF-α in the endotoxin vs. placebo condition were larger in the younger group as compared to the older group (*p’s*<.001). For T2-T0 change scores, there were significant condition by age group interactions for both change in IL-6 (*b*=-1.31, *SE*=0.31, *p*<.001; 95% CI[-1.91, -0.70]) and change in TNF-α (*b*=-0.79, *SE*=0.20, *p*=.0002; 95% CI[-1.20, -0.39]), such that increases in IL-6 and TNF-α in the endotoxin vs. placebo condition were larger in the younger group as compared to the older group (*p’s*<.001).

**References**

1. Eisenberger NI, Inagaki TK, Mashal NM, Irwin MR (2010): Inflammation and social experience: An inflammatory challenge induces feelings of social disconnection in addition to depressed mood. *Brain, Behavior, and Immunity* 24: 558–563.

2. Eisenberger NI, Berkman ET, Inagaki TK, Rameson LT, Mashal NM, Irwin MR (2010): Inflammation-induced anhedonia: endotoxin reduces ventral striatum responses to reward. *Biological Psychiatry* 68: 748–754.

3. Elin RJ, Wolff SM, McAdam KPWJ, Chedid L, Audibert F, Bernard C, Oberling F (1981): Properties of reference escherichia coli endotoxin and its phthalylated derivative in humans. *Journal of Infectious Diseases* 144: 329–336.

4. Krabbe KS, Bruunsgaard H, Qvist J, Hansen CM, Møller K, Fonsmark L, *et al.* (2001): Hypotension during endotoxemia in aged humans: *European Journal of Anaesthesiology* 18: 572–575.

5. Suffredini AF, Hochstein HD, McMahon FG (1999): Dose‐related inflammatory effects of intravenous endotoxin in humans: evaluation of a new clinical lot of escherichia coli O:113 endotoxin. *J INFECT DIS* 179: 1278–1282.

6. Suffredini AF, Hochstein HD, McMahon FG (1999): Dose-related inflammatory effects of intravenous endotoxin in humans: evaluation of a new clinical lot of Escherichia coli O:113 endotoxin. *J Infect Dis* 179: 1278–1282.

7. Suffredini AF, Noveck RJ (2014): Human endotoxin administration as an experimental model in drug development. *Clin Pharmacol Ther* 96: 418–422.
